# Quality and accuracy of complementary feeding information on Instagram: A cross-sectional content analysis study

**DOI:** 10.1371/journal.pone.0359543

**Published:** 2026-10-21

**Authors:** Alysia Rafter, Emerald S. Proctor, John J. Chen, Marie Conway, Marie K. Fialkowski

**Affiliations:** 1 School of Biological Sciences, Technological University Dublin, Grangegorman, Dublin, Ireland; 2 Trinity College Dublin, The University of Dublin, College Green, Dublin, Ireland; 3 Department of Quantitative Health Sciences, John A. Burns School of Medicine, University of Hawaiʻi, Honolulu, United States of America; 4 Nutrition Support Shared Resource, University of Hawaiʻi Cancer Center, Honolulu, United States of America; United Arab Emirates University, UNITED ARAB EMIRATES

## Abstract

Instagram is a social media platform commonly used by parents and caregivers to source complementary feeding advice, yet the reliability of the information provided is unknown. This study aimed to evaluate the quality and accuracy of complementary feeding information disseminated on Instagram, a global digital communication platform, and to assess its alignment with World Health Organization (WHO) guidelines. A cross-sectional content analysis study was conducted on Instagram in March 2025. 13 complementary feeding-related hashtags were used to identify relevant posts. Quality was evaluated using a modified version of the Principles for Health Information on Social Media (PRHISM) tool, which applied 11 relevant principles to evaluate the digital health content. Alignment with WHO recommendations for timing of introduction and food groups were used to assess accuracy. 650 Instagram posts were identified. Following screening, 302 posts were evaluated for quality and accuracy. Of the 103 posts that mentioned when to introduce complementary foods, 93.2% recommended introduction at 6 months, in line with WHO recommendations. Among the 268 posts addressing food types, 52.6% promoted consumption of all three WHO-recommended food groups. Overall, information quality was classified as mediocre according to the PRHISM criteria, with most posts (93.0%) scoring between 26 and 50 out of 100. The lowest scoring PRHISM domains were attribution, supplementary information, and the presentation of risks and benefits. Instagram, a widely accessed channel for health advice, provides complementary feeding information that varies in accuracy and is of mediocre quality. While accurate for the timing of complementary food introduction when compared to WHO guidelines, the accuracy of the types of foods recommended, and the quality of information provided, are more variable. These findings underscore the need for digital health policies, content regulation, and professional engagement to improve the quality and accuracy of nutrition-related information on social media and promote equitable, evidence-based caregiving practices.

## Introduction

In recent years, social media platforms have become dominant sources of public health information [1]. Instagram is a platform that provides a range of health-related content and includes advice on infant nutrition and feeding practices [2–4]. With its quick, digestible format, Instagram allows health messages to reach a wide audience; however, this open accessibility also poses challenges related to the accuracy and quality of posts due to personal biases, anecdotal experiences and commercial interests [5].

Parents and caregivers retrieve infant nutrition advice from a variety of sources such as family, friends and healthcare providers [6–8]. Social media is also an increasingly important resource for parents and caregivers to access infant feeding information [2,3]. A 2020 cross-sectional survey of parents of children aged 0–18 years in the US revealed that 96% of participating parents used social media, with 68% using these platforms for health information specifically [9]. The usage of Instagram is increasing and it is particularly prevalent among mothers and individuals under 40 [10], so can influence decision-making among young mothers in particular.

Complementary feeding refers to the transition of infants from exclusive breastfeeding or formula feeding to the introduction of other foods [11]. Commencing intake of other foods is necessary to accommodate the infant’s changing requirements and physiological maturation [12]. It reduces the risk of growth faltering, malnutrition, developmental delays of feeding skills, and deficiencies of micronutrients such as iron and zinc. Complementary feeding methods can also influence lifelong flavor preferences and dietary habits that will impact long-term health [13], and is a key area of nutrition to address.

The World Health Organization (WHO) provides specific recommendations on when and how to introduce complementary foods to ensure optimal growth and development. WHO recommends the introduction of complementary foods at around 6 months of age, alongside breastfeeding and a varied diet with three recommended food groups (1. animal source foods (meat, fish, eggs, dairy), 2. fruits and vegetables, and 3. pulses, nuts, and seeds) [12]. However, the extent of which publicly accessible content on Instagram aligns with these guidelines remains unknown.

Previous studies have examined the quality and accuracy of general nutrition information on social media and have found the information to be inconsistent and unreliable [14–17]. Although data is available on general nutrition information, to date no studies have assessed the quality of complementary feeding information on social media and whether this information aligns with official recommendations. Engagement rates and use of social media for infant health advice have been investigated [2,3,18–22], but no studies have systematically analyzed the quality and accuracy of this content. The current study aims to evaluate the quality and accuracy of complementary feeding information disseminated on Instagram, using the PRHISM digital health tool and WHO guidelines. A secondary objective of this study was to determine whether the professional background of account holders was associated with the quality and accuracy of the information provided. The study provides important insights into the credibility of Instagram as a source of complementary feeding information.

## Materials and methods

### Study design

This was a cross-sectional content analysis study of Instagram posts related to complementary feeding, guided by the Strengthening the Reporting of Observational Studies in Epidemiology (STROBE) checklist [23].

### Ethical considerations

This study was exempt from formal ethics review due to the public nature of the data. To maintain ethical standards, identifiable details of Instagram accounts were excluded from analysis and reporting.

### Eligibility criteria

To be included, posts had to include information on when to introduce complementary feeding and/or the types of foods to introduce or avoid. The content must have been published within the last five years and written in English. The posts could not contain video or video-related content. Posts that did not meet the inclusion criteria were excluded from analysis.

### Data collection

A new Instagram account was created on a desktop’s incognito mode to avoid algorithmic bias [15,16]. Hashtags related to complementary feeding were collected through an exploratory search using the search bar for the terms *‘complementary feeding’, ‘starting solids’,* and *‘weaning’.* Posts generated from these searches were reviewed, and any hashtags that appeared in the captions were documented. For each hashtag, the number of associated posts was recorded and used to rank them in order of popularity. Hashtag collection continued until no newly identified hashtags had a higher post count than those already listed [24].

The 13 most frequently used hashtags (each with >200,000 uses), such as #babyledweaning, #firstfoods, and #startingsolids, were selected to identify complementary feeding-related posts (full list of hashtags used available in S1 Table). The top 50 posts for each hashtag were collected over a 5-day period between 9^th^ and 13^th^ of March 2025. This resulted in an initial dataset of 650 posts, consistent with that of similar social media content analysis studies [25–27]. Posts were manually screened for eligibility and duplicates based on matching captions and dates of publication. Content analysis was conducted on the images and captions of eligible posts. As has been done in previous studies [28–30], the content analysis focused solely on the information contained within the posts themselves, without examining account profiles or bios. However, any profession stated within the post content was recorded.

### Content analysis

The recommended age for introducing complementary foods and the types of foods to introduce or to avoid were documented as applicable (Table 1). Any food mentioned that could be categorized into three WHO-recommended food groups (1. animal-source foods (meat, fish, eggs, dairy), 2. fruits and vegetables, and 3. pulses, nuts, and seeds) were recorded. Within each food group category, individual food items were documented; for instance, banana was placed under ‘fruit’ and yoghurt under ‘animal-source foods.’ Any commentary on food categories to avoid when complementary feeding was documented, including foods high in salt and sugar, and sugar sweetened beverages.

**Table 1:** Criteria to assess the accuracy of complementary feeding information on Instagram posts according to WHO* guidelines.

Criteria for Accuracy [11]	Description
Timing of Introduction of complementary foods (months of age)	Does the post recommend introducing complementary foods at 6 months of age?
Recommended Food Groups	Does the post mention any of the following food groups: • Animal-source foods (meat, fish, eggs, dairy) • Fruits and vegetables • Pulses, nuts, and seeds
Foods to avoid	Does the post discourage consumption of:• Foods high in sugar, and/or• Foods high in salt, and/or• Sugar-sweetened beverages

* WHO= World Health Organization

The professional background of account holders (dietitian, doctor, other, or unable to confirm) was noted. In this context, ‘account holders’ refers to the individuals who posted complementary feeding content on Instagram. The terms ‘dietitian’ and ‘doctor’ were recorded as those who self-identified as dietitians or medical doctors; however, professional credentials could not be independently verified due to the limitations of confirming qualifications on social media [31]. ‘Unable to confirm’ referred to cases where the account holder’s professional background could not be identified from the post. Accounts that did not fall into these categories were classified as ‘other’, such as individuals in non-healthcare professions.

### Quality and accuracy evaluations

Quality was assessed using a modified version of the Principles for Health Information on Social Media (PRHISM) tool, which was a structured framework designed to evaluate the quality of public health information on social media [32]. Unlike traditional website-focused tools, PRHISM addresses the unique characteristics of social media content [33,34]. Developed via a modified Delphi process [35], the tool outlines 13 quality principles relating to aspects such as clarity, accessibility, and the provision of evidence-based information. For this study, Principle 1 (Authorship) and Principle 2 (Authoritativeness) were excluded, as they pertain to information in account bios, which were not evaluated. The current study focused solely on assessing the quality of content within the posts themselves. The remaining 11 principles were assessed: *Action-Oriented Content, Financial Disclosure, Attribution, Balance & Justifiability, Risks & Benefits, Privacy, Complementary Information, Referrals & Support, Readability & Comprehensibility, Accessibility,* and *Use of Images*. A comprehensive breakdown of the PRHISM tool and its principles are described elsewhere (S2 Table) [15,32]. Each principle was scored on a 5-point Likert scale, based on how well the content aligned with the principle. The overall PRHISM score was calculated as a percentage using total score/possible score x 100. Scores range from 0 to 100, with higher scores indicating higher content quality. Quality ratings were categorized into four levels: scores from 0 to 25 as poor, 26–50 as mediocre, 51–75 as good, and 76–100 as excellent [15].

Accuracy was evaluated based on alignment with WHO-recommended food groups (Table 1). Yes/no labels were input based on whether a food group was mentioned and/or the timing mentioned was correct or not. Total PRHISM scores were used to identify the overall quality category.

### Coding and reliability

A structured coding framework, with a set of coding rules was created to improve consistency and minimize conflicts [36]. To assess inter-coder reliability, a sample of every 10^th^ post (10% of posts) was independently coded by a second researcher as has been utilized in other content analysis studies [37]. Krippendorff’s alpha was used to determine the level of agreement between coders. Its values range from 0 to 1, with 1 indicating perfect reliability and 0 suggesting the absence of reliability [38]. Disagreements were resolved through discussion until consensus was reached. Based on the patterns of disagreement and the consensus, the remaining 90% of posts were updated to reflect the new understanding through the consensus building process.

### Data analysis

The dataset was exported from Google sheets to Microsoft Excel for preliminary data cleaning, before being imported into IBM SPSS Statistics (Version 29.0) (SPSS, IBM, USA) for statistical analysis. Within SPSS, a combination of descriptive and inferential statistical analyses was completed. Descriptive statistics, such as frequencies and percentages, were used to describe categorical data, such as PRHISM scores and alignment with WHO accuracy criteria. Mean ± standard deviations [SDs] were reported for individual PRHISM principles, to provide insight into content strengths and weaknesses. Chi-Square tests of independence and Fisher’s Exact Tests were conducted to assess associations, depending on expected cell sizes. Statistical significance was defined as *p* value <0.05.

## Results

Following screening, a total of 302 posts met the criteria for inclusion in the analysis (Fig 1). Inter-coder reliability tests revealed a Krippendorff’s alpha value of 0.799, indicating a high level of agreement between coders and acceptable reliability for content analysis [38].

**Fig 1:**
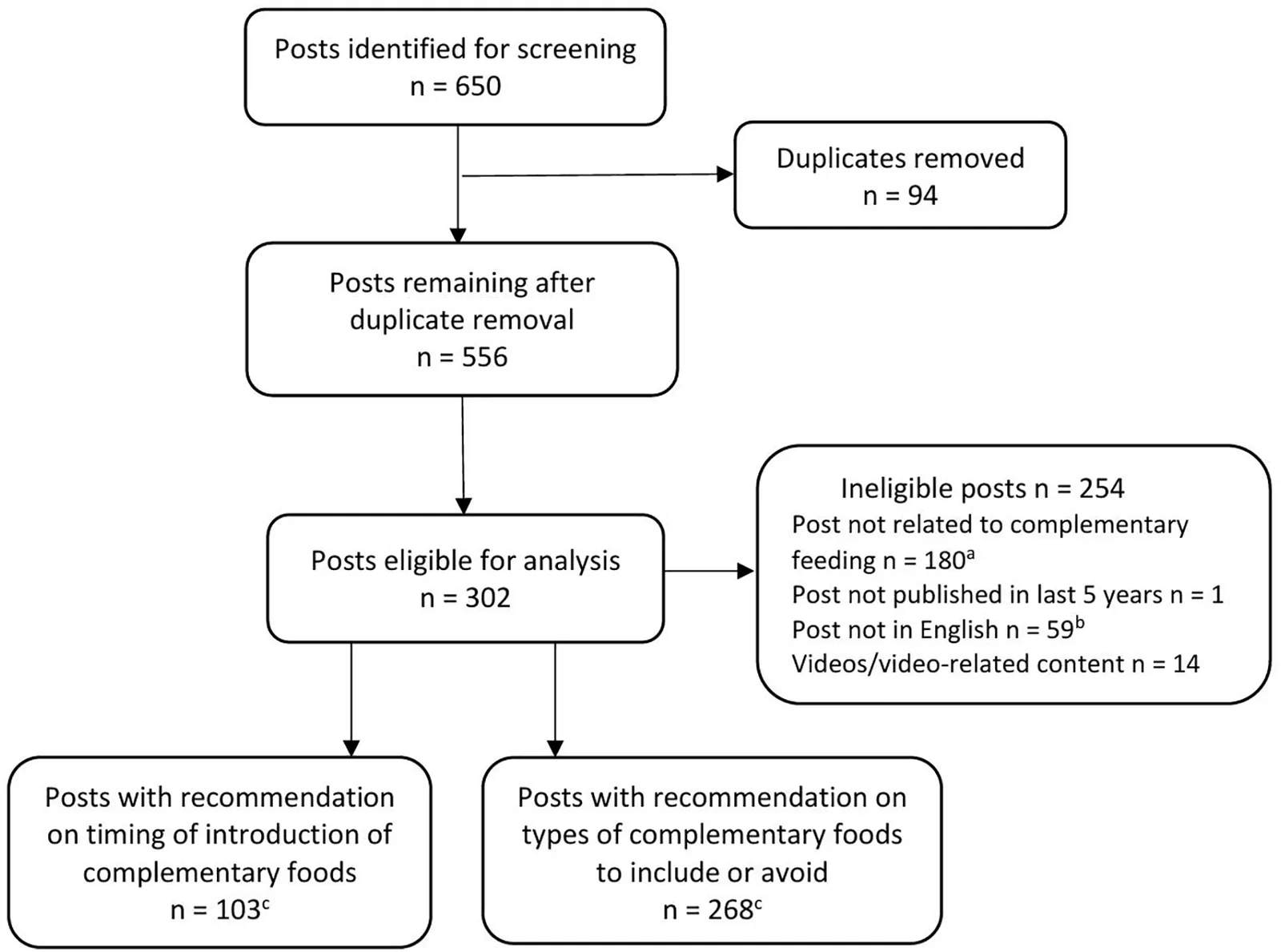
Flowchart of the sample selection process.

Over a third of the posts (n = 103, 34.1%) included information on when to introduce complementary feeding, while most of the posts (n = 268, 88.7%) mentioned types of complementary foods to introduce. The professional status of the majority of account holders (83.4%) could not be confirmed from the posts alone, however a small proportion identified as dietitians or doctors. The ‘Other’ subgroup (n = 3) was included in descriptive statistics; however, due to its small sample size, it was not included in subgroup statistical analyses. The ‘Other’ subgroup included n = 2 nutritionists and n = 1 parenting coach.

^a^ Posts not in English included Arabic, German, Portuguese, and Spanish ^b^ Posts excluded for not relating to complementary feeding included content on maternal nutrition, feeding beyond 23 months of age, or other infant care topics not specific to feeding ^c^ A sample of posts (n = 69) included recommendations for both timing of introduction and types of foods, and were accounted for in both categories

### Quality of complementary feeding information shared on Instagram

During analysis, one of the 11 PRHISM principles was excluded from scoring (Principle 8 [Privacy]) due to the complete lack of applicable content across all posts. Quality scores were therefore calculated based on the remaining 10 principles.

The majority of posts were classified as mediocre, with few achieving a ‘Good’ quality rating. No posts were identified as ‘Excellent’ quality (Table 2). A Fisher’s Exact test revealed a statistically significant association between account type and PRHISM category χ^2^(6, N = 302) = 33.42, *p* < 0.001. Dietitians were identified as the primary contributors to high-quality content, with the highest proportion of ‘Good’ posts. Other account types, particularly unverified accounts, presented less in the ‘Good’ category.

**Table 2:** Principles for Health Information on Social Media (PRHISM) quality category based on account type.

Account Type*	Total, N (Col%)	Poor n (Row%)	Mediocre n (Row%)	Good n (Row%)	Excellent n (Row%)
**All accounts**	302	15 (5.0)	281 (93.0)	6 (2.0)	0 (0.0)
**Dietitian**	35 (11.6)	0 (0.0)	30 (85.7)	5 (14.3)	0 (0.0)
**Doctor**	12 (4.0)	0 (0.0)	12 (100.0)	0 (0.0)	0 (0.0)
**Unable to confirm**	252 (83.4)	15 (6.0)	236 (93.7)	1 (0.4)	0 (0.0)
**Other**	3 (1.0)	0 (0.0)	3 (100.0)	0 (0.0)	0 (0.0)

*Account type based on self-identification of credentials

When separated into the individual principles, out of a total score of 4, the lowest mean scores presented for complementary information, attribution, and associated risks and benefits. Three principles had a mean score of two or more (Action-orientated, Accessibility, Images) (Table 3).

**Table 3:** Mean (± SD) quality scores for complementary feeding related Instagram posts across modified PRHISM principles.

PRHISM Principle*	Mean ± Standard Deviation
3. Action-orientated	3.6 ± 0.6
4. Financial disclosure	1.5 ± 1.0
5. Attribution	0.2 ± 0.8
6. Balance & justifiability	1.4 ± 0.6
7. Risks & benefits	0.9 ± 1.1
8. Privacy	Not Applicable (N/A)
9. Complementary information	0.1 ± 0.7
10. Referrals & support	1.5 ± 1.5
11. Readability & comprehensibility	1.6 ± 1.6
12. Accessibility	2.0 ± 1.4
13. Images	4.0 ± 0.3
Overall score	34.7 ± 6.3

*Principle 1 and 2 were excluded as they related to account bios which were not evaluated

### Accuracy: Timing of introduction of complementary foods

The overwhelming majority of posts recommended introducing complementary foods at 6 months of age, aligning with WHO guidelines (Table 4). All of the posts from dietitians recommended introduction at 6 months of age. Deviations from the WHO guideline came primarily from unverified accounts and one doctor account (Table 5)

**Table 4:** Overall accuracy of complementary feeding–related Instagram posts according to World Health Organization (WHO) guidelines.

Accuracy criteria	WHO complementary feeding guideline	Total posts, N	Accurate posts, n (%)	Inaccurate posts, n (%)	P value
Timing of introduction	Introduction at 6 months	103	96 (93.2)	7 (6.8)	0.001
Types of foods: to introduce	All 3 WHO-recommended food groups	268	141 (52.6)	127 (47.4)	0.001
Types of foods: to avoid	Avoid high sugar foods	268	29 (10.8)	239 (89.2)	
	Avoid high salt foods		22 (8.2)	246 (91.8)	
	Avoid sugar-sweetened beverages		4 (1.5)	264 (98.5)	

**Table 5:** Accuracy of complementary feeding–related Instagram posts according to World Health Organization (WHO) guidelines by account type.

		Accuracy based on Account type				
Accuracy criteria	WHO complementary feeding guideline	‘Dietitian’ n (%)	‘Doctor’ n (%)	‘Unable to Confirm’ n (%)	‘Other’ n (%)	P value
Timing of introduction	Introduction at 6 months	15 (100.0)	5 (83.3)	74 (93.6)	2 (100.0)	0.001
Types of foods to introduce	All 3 WHO-guided food groups	28 (87.5)	6 (60.0)	106 (47.5)	1 (33.3)	0.001
Types of foods to avoid	Avoid high sugar foods	5 (14.2)	2 (16.7)	21 (8.3)	1 (33.3)	
	Avoid high salt foods	6 (17.1)	1 (8.3)	15 (5.9)	0 (0.0)	
	Avoid sugar-sweetened beverages	0 (0.0)	0 (0.0)	4 (1.6)	0 (0.0)	

A Chi-Square test of independence revealed a statistically significant association between account type and the month recommended for introducing complementary foods (χ^2^(4, N = 103) = 17.84, *p* = 0.001). Dietitians had the highest accuracy for timing of introduction relative to other account types.

### Accuracy: Types of complementary foods to introduce

Over half of posts with information on foods to include mentioned all 3 food groups recommended by WHO. Most of the remaining posts mentioned 2 of the 3 groups (Table 4). There was a significant association between account type and recommended food groups to introduce (χ^2^(6, N = 268) = 22.25, *p* = 0.001). Dietitians most frequently mentioned all three WHO-recommended food groups (Table 5). The most common food group to recommend was fruit and vegetables (96.6%), followed by animal products (85.4%), and then other plant sources (63.8%). The most frequently recommended individual foods included bananas (45.5%), avocados (42.1%), and apples (18.0%) for fruits; broccoli (37.3%), sweet potatoes (32.8%), and carrots (29.5%) for vegetables; eggs (51.5%), yoghurt (34.3%), and chicken (30.6%) for animal products; and nuts (34.7%), peas (28.4%), and seeds (27.6%) for other plant sources.

### Accuracy: Types of food to avoid

Advice to avoid certain foods appeared infrequently across all account types, with avoidance of high-sugar foods, high-salt foods, and sugar-sweetened beverages mentioned in approximately 20% of all posts (Table 4).

## Discussion

This was the first known study to investigate the quality and accuracy of complementary feeding information shared on Instagram. It provides important insight into information on Instagram, a resource of increasing relevance for health and nutrition advice, but largely under researched in the area of complementary feeding.

Overall, Instagram posts on complementary feeding were of mediocre quality, i.e., ‘acceptable but not good enough’ [39]. The absence of any posts classified as excellent quality highlights the need for improvement in the nutritional information shared on this platform. These findings reflect those of broader nutrition studies that have highlighted the presence of low to moderate quality information over a range of nutrition topics on social media and reiterate that current information quality is suboptimal [16,17]. An evaluation of posts and accounts of Australian influencers, which also used the PRHISM tool, found the quality to be low-mediocre and also had no posts of “excellent” quality [15]. This indicates the presence of substandard quality information over a range of nutrition topics. This issue is particularly important in terms of infant nutrition, as appropriate feeding practices are fundamental to growth in formative years and minimizes the risk of developing chronic diseases like obesity, micronutrient deficiencies and poor long-term feeding habits [11,40,41].

Many posts reviewed in the study had poor evidence basis for claims, a lack of acknowledgement of the value of healthcare providers in the provision of health advice, and an absence of a robust outline of the risks and benefits of any advice given. Addressing these issues has the potential to enhance the reliability of feeding advice available on Instagram and ultimately support better infant feeding practices for those who source health information on the platform [42].

In terms of accuracy, the findings indicate high levels of accuracy are present for the timing of introduction of complementary foods when compared to WHO guidelines. Posts recommending types of foods to introduce were less accurate, with only half being in line with WHO criteria. Thus, timing of introduction of complementary foods is generally well understood and accurately conveyed across Instagram, and the dietary diversity of foods recommended is less consistently aligned. Public health messaging on social media should aim to ensure accuracy across all aspects of complementary feeding.

Multiple studies have highlighted the inaccuracies present in health and nutrition information on social media, including the dissemination of incorrect information, non-scientific health claims and sensationalized stories [16,43]. However, other more specific and well-defined topics have identified high levels of accuracy, such as in cancer-related information [44], endometriosis [45] and healthy eating blogs [46]. Given the absence of research evaluating the accuracy of complementary feeding information on social media, no direct comparisons can be made with existing literature on this topic. However, the findings suggest that specific, well-defined topics can have higher levels of accuracy than broader nutrition content. Overall findings are mixed and there is a lack of consistency in the degree of accuracy of nutrition-related content on social media.

Limited information was available regarding foods to avoid during complementary feeding, with only a small proportion of posts referencing the avoidance of added salt, sugar, or sugar-sweetened beverages. While this absence does not imply that the information presented was incorrect or that these topics were intentionally excluded, it highlights a notable gap in messaging. Greater emphasis should be placed on communicating the importance of avoiding these food items to support the development of healthy eating behaviors from an early age [42], particularly as high intake of sugar and salt can lead to increased risk of certain cardiovascular and cardiometabolic diseases in adulthood [47–49].

Posts from those who identified as dietitians received higher scores in terms of quality and accuracy overall, followed by doctors and the accounts categorized as ‘unable to confirm’. This suggests that the information provided by healthcare professionals has the highest quality and accuracy and highlights the importance of acknowledging account holders’ professional backgrounds. It aligns with the opinions of social media users, who have been seen to trust dietitians more than any other source of information [50]. However, despite dietitians posting higher quality content on average, the overall quality of their posts was not high enough to be considered excellent, indicating the need for further improvement for all account holders that are sharing information for public use.

In terms of both quality and accuracy, the lack of regulation of content posted on Instagram has impacted its ability to be a reliable source of information, despite the fact that it is already used by many for advice on health and nutrition and has been observed to have an impact on dietary behavior [1–3,51]. There is scope for its use as a nutrition resource, but additional standards, including policies and/or regulations, are needed to improve the consistency of advice available. The development of transparent guidelines by social media platforms on how to evaluate sources of information is needed to identify credible sources. Use of quality tools like PRHISM, and official guidance, like WHO guidelines, could be helpful to guide this improvement for those who share content online. Additionally, a system of ‘spot checking’ on Instagram, where content shared is monitored and managed to ensure it is up-to-standard and not disseminating inaccurate information would improve the proportion of reliable information available [5].

### Strengths

This is the first known study to provide information on the quality and accuracy of complementary feeding information on social media platforms such as Instagram and therefore addresses a gap in the literature. This study adhered to the STROBE checklist for transparent reporting, implemented a standardized codebook, and assessed intercoder reliability. It had a structured assessment method, with the use of a social-media-specific quality assessment tool, PRHISM, to evaluate the quality of the health information available, and the implementation of official WHO guidelines to evaluate accuracy.

### Limitations

The study also had several limitations. Firstly, the investigation was completed on Instagram and did not explore the quality and accuracy of complementary feeding information on other platforms. Instagram is an increasingly relevant and popular social media platform [52,53], and focus on this allowed for the provision of a high degree of specificity. Further research is warranted to determine how these findings apply to social media as a whole. Secondly, by focusing solely on English-language static posts and excluding video content, the findings may not reflect the full spectrum of complementary feeding information available to caregivers, particularly in non-English speaking communities. Future studies could incorporate these aspects to provide additional insights.

Finally, this study focused solely on the content of individual Instagram posts, without analyzing account profiles or external links to verify credentials. Consequently, most accounts were categorized as ‘unable to confirm’ in terms of professional status, which may have underestimated the number of qualified professionals. While this reflects typical user interactions with Instagram posts, it limited assessment of source credibility. Future research could incorporate account-level analysis to further classify those who share content and explore the link between professional status and post quality.

## Conclusion

Complementary feeding information on Instagram is of variable accuracy and overall mediocre quality, and has notable deficiencies in referencing, recognition of personalized healthcare needs, and acknowledgement of associated risks and benefits. Improving content quality can be supported through public guidance on identifying credible sources, increased engagement from healthcare professionals, and platform-level screening for inaccurate information. This study contributes to the limited body of research exploring the role of social media as a resource for complementary feeding information. As social media increasingly shapes parental decision-making, ensuring the dissemination of evidence-based and trustworthy advice is essential to support optimal complementary feeding and improve infant health outcomes.

## Supporting information

S1 Table Hashtag selection process. (TIF)

S2 Table Principles for Health Information on Social Media (PRHISM) scoring tool. (TIF)
