# Supplementary material for: Quality and accuracy of complementary feeding information on Instagram: A cross-sectional content analysis study
Source: PLoS One. 2026 Oct 21;21(10):e0359543. doi: 10.1371/journal.pone.0359543 (PMC13686500; doi:10.1371/journal.pone.0359543)
Supplement: Supplementary file 1 [file pone.0359543.s004.docx]

**Manuscript Title:** Quality and accuracy of complementary feeding information on Instagram: a cross-sectional content analysis study

**Manuscript ID:** PONE-D-26-01320

Dear Academic Editor and Reviewer,

We sincerely thank you for your careful evaluation of our manuscript and for your constructive comments. We appreciate the time and effort devoted to reviewing our work. We have carefully considered all comments and revised the manuscript accordingly. A detailed, point-by-point response is provided below. All changes have been highlighted in the marked-up version of the manuscript.

**Comment 1:** *The manuscript is well-written. The objectives, methods, and findings are clearly narrated.*

**Response:** We thank the reviewer for their positive feedback and appreciation of our work.

**Comment 2:** *Based on my experience of publishing with PLOS ONE, the data availability policy requires raw datasets so that the findings can be reproduced. Therefore, is it possible to share the social media contents, which were analyzed?*

**Response:** We thank the reviewer for this important comment. The study analyzed publicly available Instagram posts. However, the original post content (including screenshots of posts and captions) cannot be deposited in a public repository because of copyright and platform-related restrictions. To facilitate reproducibility, we have deposited the de-identified coded dataset used for the analyses, together with the study codebook describing all variables, in a public repository and have updated the Data Availability Statement accordingly.

**Comment 3:** *In Tables 4 and 5, please include the p-values as narrated in the result section.*

**Response:** Thank you for this suggestion. We have revised Tables 4 and 5 to include the corresponding p-values reported in the Results section, improving consistency between the text and tables.

We once again thank the Academic Editor and Reviewer for their thoughtful comments, which have helped improve the quality and clarity of our manuscript. We hope that the revised manuscript satisfactorily addresses all concerns and is now suitable for publication in PLOS ONE.
